# Role of imaging in third aortic valve implantation: TAV-in-TAV-in-SAV

**DOI:** 10.1007/s10554-024-03175-y

**Published:** 2024-07-06

**Authors:** Mi Chen, Jonathan M. Michel, Barbara E. Stähli, Felix C. Tanner, Albert Markus Kasel

**Affiliations:** 1 Department of Cardiology, University Hospital Zurich, Zurich, 8091 Switzerland; 2 Faculty of Medicine, University of Zurich, Zurich, Switzerland

**Keywords:** Transcatheter aortic valve implantation, TAVI, Redo-TAVI, Valve-in-valve, Valve-in-valve-in-valve

## Abstract

Coronary obstruction remains a primary concern for redo transcatheter aortic valve implantation (TAVI) due to supra-annular leaflets. Hereby, we present two valve-in-valve-in-valve cases, initially incorporating a surgical valve implanted to clarify our concept that the surgical valve serves to safeguard against the coronary ostium obstruction.

An 83-year-old man underwent surgical aortic valve replacement (SAVR) using a 25-mm Magna Ease valve (Edwards Lifesciences) 10 years ago and 29-mm Evolut Pro (Medtronic) valve-in-valve transcatheter aortic valve implantation (TAVI) 3 years ago due to prosthetic valve regurgitation. Pre-TAVI computed tomography (CT) showed a low left main (LM) at 8.8 mm, right coronary artery (RCA) at 14.8 mm, and the sinotubular junction (STJ) at 23.6 mm. Compared to a 26-mm SAPIEN (Edwards Lifesciences) simulation with a < 3 mm valve-to-sinotubular-junction (VTSTJ) distance (Panel A), a 23-mm SAPIEN was chosen due to its upper frame sitting below the STJ (Panel B). Notably, the Evolut Prosthesis has expanded the leaflets of the pre-existing surgical valve, dressing a higher “neo-skirt” at the surgical valve height. Therefore, surgical valve prevents coronary obstruction with the same height implant of the 3rd valve: (1) no more overexpansion to approach the coronary ostium; (2) no higher neo-skirt; (3) leaflet overhang is compromising given that prosthetic regurgitation rather than stenosis; (4) predicted indexed effective orifice area (0.74) (Panel J). A 23-mm SAPIEN was placed “outflow to outflow” with the surgical valve, resulting in the same-height deployment (Panel F and G).

A 79-year-old man underwent a SAVR with a 25-mm FreeStyle Root (Medtronic) 14 years ago and a 29-mm Evolut Pro TAVI 6 years ago. Pre-TAVI CT for showed an elongated left ventricular outflow tract (LVOT) (Panel C), due to a 3-mm inner cloth height, which may allow a deeply implanted Evolut valve (Panel D). Despite the supra-annular design, the commissural plane was only 10 mm above the surgical annulus, indicating a low risk of coronary obstruction (Panel E). A 23-mm SAPIEN inflow was aligned with node 3 of the Evolut Pro prosthesis for deployment (Panel H), with “flare the outflow”(1,2) technique to ensure adequate anchoring (Panel I and K). How to manage a multi-TAV implant remains the key challenging for young patients.

**Figure:**
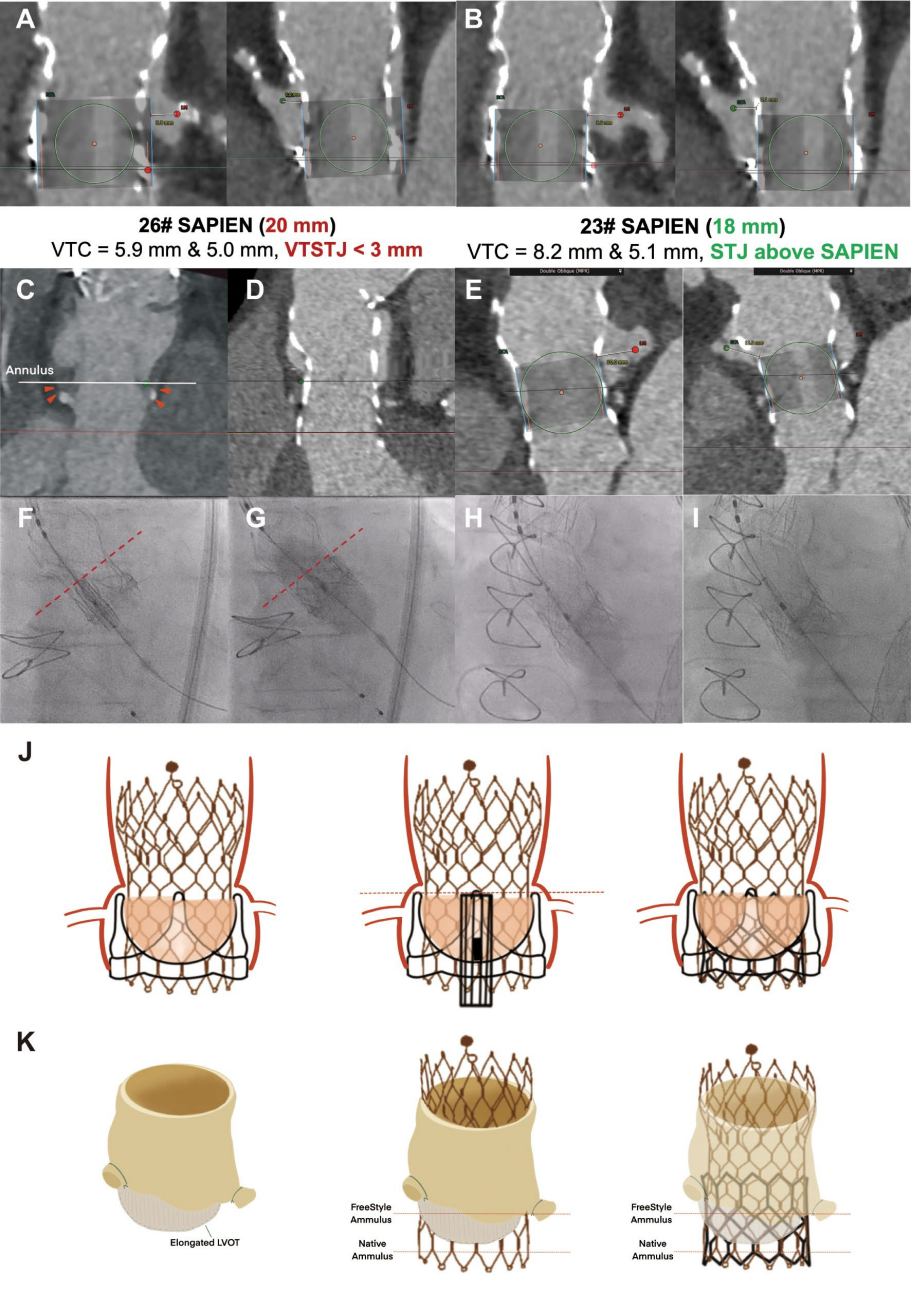

## Data Availability

Data can be required from corresponding author.

